# Bridging the Care Gap: Integrating Family Caregiver Partnerships into Healthcare Provider Education

**DOI:** 10.3390/healthcare13151899

**Published:** 2025-08-04

**Authors:** Jasneet Parmar, Tanya L’Heureux, Sharon Anderson, Michelle Lobchuk, Lesley Charles, Cheryl Pollard, Linda Powell, Esha Ray Chaudhuri, Joelle Fawcett-Arsenault, Sarah Mosaico, Cindy Sim, Paige Walker, Kimberly Shapkin, Carolyn Weir, Laurel Sproule, Megan Strickfaden, Glenda Tarnowski, Jonathan Lee, Cheryl Cameron

**Affiliations:** 1 Department of Family Medicine, Faculty of Medicine & Dentistry, University of Alberta, Edmonton, AB T6G 2T4, Canada; jasneet.parmar@albertahealthservices.ca (J.P.); lesley.charles@albertahealthservices.ca (L.C.); gtarnowski20@gmail.com (G.T.); 2 Rady Faculty of Health Sciences, College of Nursing, University of Manitoba, Winnipeg, MB R3T 2N2, Canada; michelle.lobchuk@umanitoba.ca; 3 Faculty of Nursing, University of Regina, Regina, SK S4S 0A2, Canada; cheryl.pollard@uregina.ca; 4 Family Caregiver, Edmonton, AB T6G 2T4, Canada; lpowell1066@gmail.com (L.P.); laurel.sproule@gmail.com (L.S.); 5 Senior Education and Equity Policy Consultancy, Edmonton, AB T6G 2T4, Canada; 6 Sim Centre, University of Alberta, Edmonton, AB T6G 2T4, Canada; 7 SPEECHified, Edmonton, AB T8A 4T7, Canada; 8 Team CarePal, Edmonton, AB T5J 1W8, Canada; cindy@teamcarepal.com; 9 Alberta Council on Aging, Edmonton, AB T5M 2T9, Canada; pmwalker16@gmail.com; 10 Faculty of Nursing, University of Calgary, Calgary, AB T2N 4L6, Canada; kimberly.shapkin@ucalgary.ca; 11 Faculty of Health Studies, Norquest College, Edmonton, AB T5J 1L6, Canada; carolyn.weir@norquest.ca; 12 Department of Human Ecology, University of Alberta, Edmonton, AB T6G 2N1, Canada; strickfa@ualberta.ca; 13 Ornge Air Ambulance, Mississauga, ON L4W 5H8, Canada; jlee@ornge.ca; 14 Department of Paramedicine, Monash University, Frankston, VIC 3199, Australia; cheryl@virtualhospice.ca

**Keywords:** caregiver, health workforce, education, healthcare providers, Kirkpatrick–Barr, evaluation, mixed methods

## Abstract

**Background**: Family caregivers are a vital yet often under-recognized part of the healthcare system. They provide essential emotional, physical, and logistical support to individuals with illness, disability, or frailty, and their contributions improve continuity of care and reduce system strain. However, many healthcare and social service providers are not equipped to meaningfully engage caregivers as partners. In Alberta, stakeholders validated the Caregiver-Centered Care Competency Framework and identified the need for a three-tiered education model—Foundational, Advanced, and Champion—to help providers recognize, include, and support family caregivers across care settings. This paper focuses on the development and early evaluation of the Advanced Caregiver-Centered Care Education modules, designed to enhance the knowledge and skills of providers with more experience working with family caregivers. The modules emphasize how partnering with caregivers benefits not only the person receiving care but also improves provider effectiveness and supports better system outcomes. **Methods**: The modules were co-designed with a 154-member interdisciplinary team and grounded in the competency framework. Evaluation used the first three levels of the Kirkpatrick–Barr health workforce education model. We analyzed pre- and post-surveys from the first 50 learners in each module using paired *t*-tests and examined qualitative feedback and SMART goals through inductive content analysis. **Results**: Learners reported a high level of satisfaction with the education delivery and the knowledge and skill acquisition. Statistically significant improvements were observed in 53 of 54 pre-post items. SMART goals reflected intended practice changes across all six competency domains, indicating learners saw value in engaging caregivers as partners. **Conclusions**: The Advanced Caregiver-Centered Care education improved providers’ confidence, knowledge, and skills to work in partnership with family caregivers. Future research will explore whether these improvements translate into real-world practice changes and better caregiver experiences in care planning, communication, and navigation.

## 1. Introduction

Family caregivers play a vital yet often unrecognized role in sustaining the healthcare system [1,2]. They provide essential physical, emotional, and logistical support to individuals living with illness, disability, or frailty, enabling many to remain at home and avoid institutional care [3,4,5]. As pressures mount to manage costs, reduce hospital stays, and support aging in place, the responsibilities of caregivers have expanded dramatically [6,7,8]. Nearly half now perform complex clinical tasks once carried out by trained professionals, such as administering medications, managing pain, operating medical equipment, and coordinating care across multiple providers [7,9,10,11]. Their contributions benefit not only care recipients but also providers and health systems, ensuring continuity of care, promoting adherence to treatment, and reducing avoidable hospitalizations [1,2].

Despite their central role, family caregivers remain at the margins of the healthcare system—underrecognized and undersupported [1,4,12]. While person- and family-centered care frameworks emphasize collaboration, caregivers often lack a defined role on the care team [13,14,15]. Health systems remain largely patient-focused, treating family involvement as a “side-of-the-desk” activity rather than a structured and supported relationship [1,5,13]. National surveys show that only 6% of caregivers for older adults with complex needs receive any form of training, and more than half report no interaction with the medical team [9,16,17,18]. When engagement does occur, it is often brief, minimally helpful, or one-directional, reinforcing a persistent disconnect between caregivers’ contributions and how healthcare providers engage with them. This lack of integration not only undermines the quality of care but also contributes to caregiver strain and isolation.

Caregivers also face structural barriers in navigating systems that are fragmented, time-constrained, and medically driven. Coordinating care, managing transitions, and advocating for family members are among the most stressful aspects of caregiving [16,19,20,21]. Caregivers frequently lack access to information, are excluded from decision-making processes, and encounter clinicians who may not fully recognize or value their insights. Providers, for their part, often report lacking the time, training, or confidence to engage caregivers meaningfully [22,23,24]. These barriers are often amplified for caregivers from marginalized or racialized communities, whose experiences may be further complicated by language differences, cultural norms, immigration status, or historical distrust of healthcare systems.

This reinforces the need for provider education that goes beyond general principles of inclusion to actively address the complexities of caregiver diversity. These challenges are further compounded by the increasing diversity among caregivers, who vary widely in age, gender, culture, language, religion, socioeconomic status, and geographic location [25,26]. While cultural competence is often framed narrowly around race and ethnicity, truly person- and caregiver-centered care requires cultural safety, humility, and responsiveness to the diverse identities and lived experiences of caregivers [27,28]. Without attention to these dimensions, provider education risks perpetuating exclusion or bias, rather than promoting inclusive, equitable care.

Emotional distress and role strain are widespread among caregivers, especially those providing intensive support for individuals with dementia, serious mental illness, or complex disabilities [9,10,29]. In 2022, 44% of caregivers reported experiencing high levels of distress [9]. As Schulz et al. [4] noted nearly a decade ago, caregivers should not be viewed merely as unpaid labor to offset healthcare costs, but as essential partners in care who need recognition, training, and support. Investing in provider education is key to reducing caregiver burden and improving outcomes for care recipients [1,30,31].

This paper addresses a persistent gap in provider education: the absence of competency-based training that prepares health professionals to engage family caregivers as care partners. Caregivers possess deep knowledge about the needs, preferences, and daily realities of those they support. However, their capacity to contribute meaningfully depends on whether providers are equipped to collaborate with them [1,31,32]. Yet most health professionals receive little or no formal instruction in identifying, communicating with, or supporting family caregivers [1,30,31]. As with other cultural and relational shifts in healthcare, education plays a critical role in reinforcing expectations, shifting behaviors, and embedding new ways of working [33,34,35,36].

Competency-based education is a key lever for this change. It supports the development of practical, relational, and reflective skills that enable providers to build effective partnerships with caregivers [33,36]. By aligning education with the realities of caregiving, health systems can foster more inclusive and responsive care practices [1,2,12,37]. As with other areas of system transformation, education is foundational to enabling behavior change and sustainable practice improvement [38,39].

### Developing Competency-Based Education to Support Caregiver Partnerships

Between 2014 and 2017, extensive stakeholder consultations across Alberta revealed a striking consensus: while caregiver support was needed in many areas, the greatest gap lay within the healthcare system itself [40,41,42,43]. Collaborating partners, including clinicians, educators, leaders, and caregivers, called for provider education that would integrate caregivers into care planning, grounded in a person-centered approach. In response, we developed the Caregiver-Centered Care Competency Framework, with six domains reflecting the knowledge, skills, and attitudes providers need to engage caregivers as partners [44,45]. We also introduced the term “caregiver-centered care” to describe person-centered care for family caregivers, aimed at supporting both their caregiving role and their own well-being [45].

Three levels of education were recommended: Foundational (for all providers), Advanced (for those with more frequent caregiver interactions or leadership roles), and Champions (for change-makers leading implementation) [45]. In late 2019, we co-designed the Foundational module with over 100 stakeholders, covering all six competency domains [34]. Since its launch in November 2020, more than 5200 providers and trainees have completed the training. A supplementary COVID-19 module addressed communication and support during public health emergencies. Evaluation results of the foundational education, published in 2022, demonstrated increases in learners’ knowledge, skills, and confidence to engage caregivers [34].

While the Foundational module was well received, experienced providers expressed interest in building more advanced skills. This paper focuses on the development and early evaluation of the Advanced Education modules, which build on foundational content to deepen practical skills. We begin by describing the co-design process, followed by an evaluation of the modules’ impact using a mixed-methods approach.

The purpose of this study was to evaluate whether advanced caregiver-centered education, co-designed with stakeholders, would improve provider knowledge, confidence, and intended practice changes in working with family caregivers. We hypothesized that providers would report increased readiness to engage caregivers as partners in care after completing the modules.

## 2. Methods

This study used a mixed-methods, interpretive description design to evaluate advanced caregiver-centered education.

### 2.1. Co-Designing Advanced Education for Caregiver-Centered Care

Co-design is a collaborative, participatory approach that actively involves stakeholders with diverse perspectives to create solutions that reflect real-world complexity [46,47,48]. The purpose of this study was to develop and evaluate advanced caregiver-centered care education using a co-design methodology, informed by complexity theory, adult learning, and competency-based education principles. We hypothesized that education developed through co-design with caregivers, providers, and educators would be more applicable, engaging, and likely to impact provider behavior and caregiver-provider partnerships.

We convened a diverse, interdisciplinary team of 154 collaborators, including family caregivers, frontline providers, healthcare leaders, educators, and instructional designers. Participants were selected based on their roles in caregiving, education, health and social care delivery, and systems leadership. Diversity in age, gender, care roles, and geographic location was prioritized. Inclusion criteria included experience in caregiving or education, and interest in improving caregiver engagement.

Participants contributed across four domains: intent, capability, experience, and design [48]. The voice of intent included individuals with decision-making authority or influence. The capability voice brought practical knowledge, access to systems, or implementation resources. The experience voice represented those directly involved in caregiving or working with caregivers. The design voice, led by The Learning Design Studio in the Faculty of Medicine at the University of Alberta, focused on curriculum development and facilitation.

Building on the six domains in the Caregiver-Centered Care Competency Framework [45] and the Foundational module [34], we co-developed six asynchronous online modules (45–60 min each). These were designed to be flexible, scalable, and suitable for either in-person or online facilitation. Each module focused on one competency domain and emphasized practical application, reflection, and caregiver inclusion.

Educational content development was guided by adult learning, constructivist, and transformative education theories [39,49,50,51]. Modules featured narratives, interactive exercises, case vignettes, caregiver-provider video reflections, and practical tools. Instructional strategies were aligned with best practices in interdisciplinary education [39,52], designed to engage learners cognitively and emotionally.

### 2.2. Co-Design Process and Educational Content Development

The co-design process unfolded through monthly or bimonthly virtual meetings held via Zoom (Zoom Video Communications, Inc., San Jose, CA, USA). Sessions began with context-setting updates and stakeholder reflections, followed by breakout room discussions focused on draft content, language, tone, and tools. Each session concluded with a full-group debrief. Recordings were transcribed and analyzed for emerging insights [53].

The development process included co-writing a fictional narrative arc—the Fawcett family—to contextualize caregiver experiences across modules. These scenarios were scripted using real examples shared by caregivers and providers in breakout rooms. The narrative helped anchor learning outcomes and create emotional resonance [54,55].

Content areas were selected based on competency framework priorities, co-design feedback, and gaps identified in foundational education. Topics included recognizing the caregiver role and work; fostering relational communication; including caregivers as partners on the care team; assessing caregivers’ needs, circumstances, and wellbeing; assisting caregivers in navigating health and community systems; and understanding providers’ roles in shifting the culture and context of care [34]. Educational content was created collaboratively by educators and learning designers with expertise in health professional education, family caregiving, and digital learning design. Modules were reviewed by multiple stakeholders, including external educators, to ensure pedagogical quality, content accuracy, and contextual relevance.

### 2.3. Evaluation Study Design

**Figure 1:**
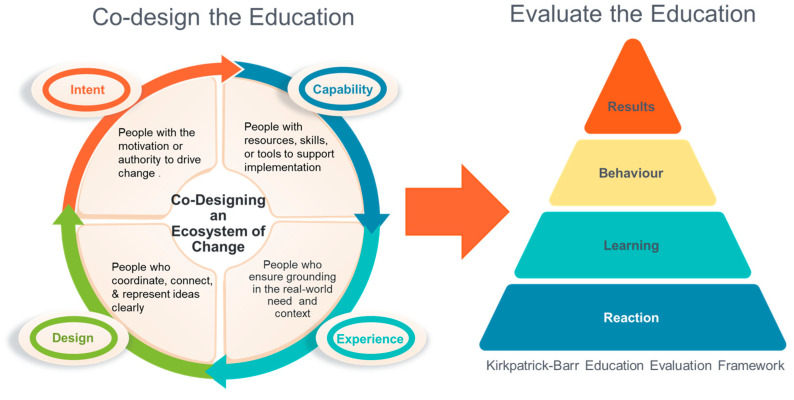
Codesigning and evaluating the education.

The evaluation aimed to assess the impact of the advanced modules on learners’ knowledge, confidence, and intended practice changes. We used an interpretive description methodology [56,57,58], supported by the first three levels of the Kirkpatrick–Barr framework [59,60]. Interpretive Description is a theoretically flexible methodology developed for applied health disciplines. It aims to generate practical, contextually relevant knowledge that supports improved practice. It is especially well-suited to mixed methods research where it can complement quantitative findings by uncovering experiential insights and complex social phenomena that are not easily captured through measurement alone [56,58]. This mixed-methods design enabled exploration of learner reactions, learning gains, and planned behavior changes (see Figure 1: Co-designing and evaluating the education).

Ethical approval was granted by the University of Alberta Health Research Ethics Board (Study ID Pro00097068). The GREET reporting guideline for education evaluation was followed [61].

#### 2.3.1. Participant Recruitment

From 30 November 2022 to 30 August 2023, health and social care providers and trainees were recruited through targeted emails, newsletters, and social media. Participation was voluntary. Informed consent was implied through survey completion. No incentives were provided. Learners could withdraw at any time by exiting the platform.

#### 2.3.2. Data Collection

Data were collected via Google Forms and Sheets. Learners completed demographic forms, pre- and post-module surveys, and submitted SMART goals reflecting intended changes. Demographics included gender, age, sector, role, care setting, and province.

- Level 1: Reactions—Four satisfaction items rated on a 7-point Likert scale assessed relevance, applicability, content engagement, and usefulness. Open-text comments captured qualitative impressions.
- Level 2: Knowledge and Confidence—Nine items aligned with the competency domains were rated pre- and post-module. These were pilot-tested with stakeholders and showed strong internal consistency (Cronbach’s alpha 0.92–0.94).
- Level 3: Planned Behavior—Learners submitted SMART goals after each module to articulate how they would apply the content in practice.

#### 2.3.3. Data Analysis

Quantitative data were analyzed using *IBM SPSS Statistics* (version 28.0; IBM Corp., Armonk, NY, USA) Descriptive statistics summarized demographics. Paired-sample *t*-tests evaluated pre-post changes in knowledge and confidence.

Qualitative data from open responses and SMART goals were analyzed using reflexive content analysis, a systematic yet flexible approach designed to reduce and describe manifest content in relation to the research questions [62,63]. Reflexive content analysis emphasizes the researcher’s reflexive engagement at each stage of analysis to ensure rigor and transparency and allows for context-sensitive interpretation while maintaining close alignment with what participants explicitly express.

Themes and patterns were developed inductively through iterative coding, using a hierarchical structure of codes, subcategories, and categories. Two researchers independently coded the data and met to refine categories and ensure conceptual clarity. Reflexive content analysis emphasis on manifest meaning was especially appropriate for capturing the concrete experiences, intentions, and learning outcomes expressed by learners. Findings were used to enrich quantitative results and provide a comprehensive understanding of how the modules influenced learner knowledge, confidence, and intentions for practice.

## 3. Results

### 3.1. Participant Characteristics

We analyzed data from the first 50 learners who completed all six advanced modules and accompanying pre-post surveys (n = 300 total module completions). As shown in Table 1, most participants identified as female (87%). Learners represented a broad range of ages, with the largest group aged 35 to 54 (50%), followed by those aged 55 to 64 (29%).

Participants came from 32 different professions and roles, reflecting the multidisciplinary nature of the types of providers who support caregivers. Most frequently reported were nurses, healthcare aides, social workers, recreation therapists, case managers, EMTs/paramedics, physicians, occupational therapists, physiotherapists, speech-language pathologists, and educators in post-secondary settings. These learners worked in a variety of care environments, including home care, long-term care, supportive living, acute care, and primary care (see Table 1: Participant characteristics).

The diversity of professional backgrounds and care settings represented in the sample highlights the broad applicability of caregiver-centered care and the need for training that meets learners where they are, regardless of discipline or sector.

### 3.2. Learners’ Reactions: Satisfaction with the Education (Level 1)

Learner satisfaction was consistently high across all six modules. Mean scores for the four key satisfaction items ranged from 6.62 to 6.80 out of 7, with a median of 7 on all items. Table 2 presents detailed satisfaction scores. Learners described education as validating and practical, noting its relevance to their daily work. Many highlighted the helpfulness of hearing directly from family caregivers and appreciated tools they could immediately integrate into practice.

### 3.3. Changes in Knowledge, Skills, and Attitudes (Level 2)

Pre-post comparisons showed statistically significant improvements (*p* < 0.001) across all competency domains. Effect sizes, based on Cohen’s d, ranged from moderate to large, with Recognizing (0.82), Fostering Resiliency (1.11), Navigating (0.98), and Changing the Culture of Care (0.85) all demonstrating large effects. Communicating (0.65) and Partnering (0.73) were in the moderate range (see Table 3 for a full summary of the results).

Open-text responses indicated that the modules helped learners connect the dots between existing practices and caregiver-centered frameworks. Some participants reported that the content helped name and organize approaches they had already been using, while others described gaining new language, strategies, and tools to strengthen partnerships with family caregivers. One learner wrote:

*“These learning videos highlighted the importance of the work that I do as a health care provider. I am confident that I provide support in many ways that these videos show—I am now able to put a name to some of the concepts and strategies utilized in my practice.”*

A recurring theme was the shift in mindset—from seeing caregivers as adjuncts to viewing them as partners whose perspectives are central to quality care. Several learners reflected on specific tools they found helpful, such as the **H**ear, **E**mpathize, **A**pologize, **R**esolve, and **T**hank (H.E.A.R.T) communication strategy; the Carer Support Needs Assessment Tool (CSNAT); and the STOP mindfulness technique. These tools were cited as practical support for enhancing caregiver communication, reducing conflict, and managing provider stress.

Learners also expressed a stronger understanding of the emotional toll on family caregivers and the importance of addressing caregiver burnout. One wrote:

*“My learning is that my job is to facilitate and have the caregiver lead conversations about their needs. this has helped me to see that the caregiver is the one who has the information.”*

Another noted

*“A wonderful course that reminds us we can work as a team and lighten the load for all involved. Everyone is a valuable team player.”*

These reflections illustrate how advanced caregiver-centered education supported learners to both validate their current approaches and take new steps toward more collaborative, respectful caregiving partnerships.

### 3.4. Planned Changes in Behaviors and Practices (Level 3)

Building on these reported learning gains, we next explored how learners translated their knowledge into planned behavioral changes. On average, 83% of respondents who submitted SMART goals were submitted. Following the Recognizing the Family Caregiver Role module, learners most frequently planned to approach caregiving situations with greater empathy and understanding. Second, they emphasized that they would recognize and affirm the contributions and sacrifices made by family caregivers in caregiving settings.

*“Over the next 6 months in the clinic, I will work to identify and document family caregivers, acknowledging their diverse roles and trajectories, to facilitate the provision of support to address unmet needs/caregiver wellness.”*

*“I would like to remember to acknowledge each caregiver’s sacrifices at the client care conferences. Writing that acknowledges the care conference questionnaire sheet, so that when the conference starts, I will remember to take a moment to acknowledge the caregiver/caregiver.”*

*“Understand, affirm, and acknowledge the unmet needs of each of the caregivers on my caseload when meeting them, at times of transition, and at reassessment.”*

In the Communicating with Family Caregivers module, learners stressed that they intended to enhance their communication skills when interacting with family caregivers through more confident and proficient communication with caregivers, ensuring that their needs and concerns were effectively addressed. Approximately one-quarter planned to use the OARS communication framework (Open-ended questions, Affirmations, Reflective listening, Summarization) to improve communication effectiveness.

*“I want to practice OARS more in my work. I will be mindful of the mnemonic while I am caring for a person and their families.”*

Notably, learners in leadership positions expressed commitments advocating for better caregiver support and communication within their organizations.

*“As a leader in an organization that deals with caregivers, I want to create a better framework—a better process—within which my team communicates with the caregivers who support loved ones in our programs.”*

Learner commitments in the Partnering with Family Caregivers Module typically included enhancing listening to family caregivers as the route to developing trust and collaborative relationships with family caregivers.

*“I plan to share information so that caregivers can be partners in care and decision making. I need to discuss how I/home care and the caregiver/client work together with my home care team. We need to practice the best way to communicate.”*

Approximately one-quarter of the learners completing the module expressed their commitment to being advocates for families and providing support during caregiver breakdowns. We introduced a tool with the mnemonic H.E.A.R.T., Hear, Empathize, Apologize, Resolve, and Thank for providers to use in deescalating tension. This tool was mentioned in almost half of the learners’ goals.

*“The family should be the central point when caring for patients. Using H.E.A.R.T. approach when tension is escalating. Encourage more staff to use H.E.A.R.T. method.”*

Health providers planned to develop learning modules, educate staff and physicians, and encourage the use of communication and de-escalation methods, such as H.E.A.R.T. among their colleagues.

*“In the next year, I want to develop learning modules for staff and physicians about how to communicate with caregivers, especially the H.E.A.R.T. process.”*

*“I have recommended this resource to leaders across my health system as a terrific example of opportunities to support the realization of an integrated people-centered health system.”*

In the Fostering Family Caregivers’ Resilience Module, learners continued to set goals of being active listeners, asking open-ended questions, and avoiding assumptions to better understand the needs and challenges faced by caregivers. We introduced the Carer Support Needs Assessment Tool-Intervention (CSNAT-I) in this module for providers to facilitate caregiver-led conversations with family caregivers about their needs. Learners added they aimed to foster resilience in family caregivers by acknowledging their strengths, coping strategies, and resources. Almost two-thirds of the learners (64%) intended to set goals with caregivers, develop action plans, and revisit these plans to ensure that caregiver needs were met over time.

*“I will continue to walk beside any caregiver and support them by allowing them to share their needs and avoid prioritizing their needs for them.”* This finding reinforces the idea that the caregiver is the captain and further nurtures resiliency. They also planned to create environments that encourage resilience by affirming caregivers’ strengths and addressing their challenges.

*“This course helped me better understand how to approach speaking about self-care and helped me understand that self-care not only lies with the caregiver but also has to be planned and executed with the help of a provider.”*

After completing the Assisting Family Caregivers to Navigate Health and Social Care Systems module, the learners committed to acting as guides when needed, supporting caregivers’ progress, and ensuring assistance and follow up as needed.

*“There is a great understanding of self-empowerment for navigation. Apart from sharing the information learned, I will make a conscious effort to follow up with family caregivers and problem solving any barriers along their way.”*

Many learners (74%) expressed the desire to continually upgrade their knowledge of the resources and services available, as well as to expand their network of contacts.

*“I would like to develop a bank of excellent contacts for caregivers and complete a visit or call with them to see their processes. This is an important area for all families with adults who need care.”*

In the Changing the Culture and Context of Care module, learners committed to uncovering unconscious biases and working on their daily attitudes and behaviors.

*“Bias, unconscious is one I need to look for. I also love the fact that one can reflect even before a situation arises and prepare oneself for responses and actions. It is important to take it one step at a time, one day of a time, and believe that the little I do daily to create a change, though not noticeable, is having an effect on the system, one person at a time.”*

Over half of the learners set goals to share Caregiver-Centered Care in their settings and with their leaders. A quarter committed to lead change in their organizations,

*“Commit to spread awareness regarding the need to pay attention to the caregiver role, as this has an immense impact on how our health system remains what it is today.”*

*“I will be an example to others of how this can work and be that leader in my setting.”*

To further illustrate the ways in which learners intended to apply their education in practice, we analyzed submitted SMART goals thematically across modules. Common themes included affirming caregivers’ contributions, improving empathic communication, and leading system-level changes. A detailed summary of SMART goal themes, descriptions, and representative examples is included in Supplementary File S4: Summary of SMART goal themes across modules.

## 4. Discussion

These findings underscore how caregiver-centered education can support healthcare and social care providers to reconceptualize family caregivers not as informal supporters, but as essential partners in care. Despite the vital roles caregivers play—from providing medical and emotional support to managing complex health needs—many remain invisible within healthcare systems [1,64]. This education aimed to change that by equipping providers with the confidence, knowledge, and practical skills needed to work in partnership with caregivers.

By improving provider-reported outcomes across six core competency domains, the Advanced Caregiver-Centered Care Education supports a cultural shift toward more inclusive, person- and family-centered care. Notably, modules with content focused on recognition of the caregiver role, relational communication, partnering, and navigation showed the greatest self-reported gains in knowledge and confidence, and they generated SMART goals focused on fostering resilience and shifting the culture of care—areas in which effect sizes were most substantial. These behavioral intentions offer early evidence of mindset change and suggest alignment between educational design and learner uptake.

These results align with prior efforts to train providers in caregiver-inclusive care, including initiatives such as the U.S. Veterans Health Administration’s web-based training on inclusive care [65]. While that intervention found no significant change in knowledge, it did increase self-efficacy and commitment to change. In our study, knowledge scores improved, and similar to Sperber [65], we too think the shift may be in provider attitudes and behavioral intentions may be more meaningful change in practice. These outcomes highlight the value of training that integrates lived experience and real-world complexity, grounded in adult learning and transformative education theory.

This work also responds to national and international calls for greater caregiver inclusion in system design and delivery [66,67,68,69]. The lack of standardized processes for caregiver identification, engagement, or support—particularly in dementia care—is widely recognized as a barrier to quality and safety [70,71,72,73]. In Alberta, we are now working with partners to embed caregiver identification into the electronic health record, building on the competencies introduced in this education.

Policy strategies are increasingly echoing the importance of provider training in caregiver engagement [66,67,68,69,74]. For example, Canada’s National Dementia Strategy [67] and provincial dementia and palliative care frameworks, e.g., 68,69], emphasize the need for provider education, caregiver identification, and recognition of caregiving as a public health priority. These strategies require operational tools—this education offers one actionable model that can be adapted across systems and professions.

Importantly, education is only one part of the solution. Structural and organizational barriers remain, including time pressures, lack of institutional incentives, and cultural norms that undervalue caregiving work. National and provincial caregiving strategies can guide this work [74,75,76,77]. Future phases of our work will focus on implementing Champion-level training to equip leaders with the tools to support sustained practice change at the team and organizational level and caregiver strategies at the policy levels.

While many providers expressed motivation to partner with caregivers, systemic pressures can limit their ability to do so. As others have found [78,79,80], clinicians often face competing priorities, including high patient loads, staffing shortages, rigid scheduling, and performance targets such as billable hours or discharge timelines. These constraints reduce opportunities to build trust, provide tailored caregiver education, or coordinate care. This points to the need not only for provider education, but also for system-level support—such as flexible scheduling, institutional recognition of caregiver engagement tasks, and integration of caregiver data into electronic health records—to enable and sustain inclusive practice.

### Limitations

This evaluation used self-report to assess knowledge, confidence, and planned behavior. While this approach cannot confirm actual practice change, it is appropriate for early-phase educational research and offers valuable insights into learner perception and intention. Knowledge testing was not prioritized due to the applied, reflective nature of the learning, and the desire to avoid reducing caregiver engagement to a checklist of facts. Future studies will include follow-up interviews and observational methods to assess long-term behavior change and impact on caregiver experience.

## 5. Conclusions

Family caregivers are essential to the health and wellbeing of people living with complex conditions, yet they remain undervalued and under-supported within our healthcare systems. This study demonstrates that caregiver-centered education—co-designed with caregivers and grounded in evidence—can play a meaningful role in shifting provider perspectives and practices. The Advanced Caregiver-Centered Care Education modules improved learners’ knowledge, skills, and confidence and encouraged the adoption of collaborative, respectful care practices that include caregivers as true partners on care teams.

By embedding real stories, practical tools, and thoughtful reflection into the curriculum, this education brought the often-invisible contributions of caregivers into clearer focus. Learners reported not only greater awareness but also a readiness to change how they engage with caregivers in their day-to-day practice. The overwhelmingly positive feedback and statistically significant learning gains suggest that even relatively short, targeted education can spark change.

Importantly, this education model does not stand alone. It is part of a broader movement toward caregiver-centered systems, aligned with national and international calls to formally recognize caregivers, support their wellbeing, and integrate their knowledge into care planning and delivery. Education is a necessary step, but it must be paired with system-level changes, including clear policies, organizational support, and consistent caregiver identification within health records.

In a patient-focused system, failing to include caregivers in care teams undermines both patient outcomes and provider effectiveness. This education offers a replicable and scalable approach to closing that gap. As we continue to evaluate outcomes and scale up Champion-level education, our hope is that this work will contribute to a culture where caregivers are not only acknowledged, but supported, equipped, and embraced as essential partners in care.

## Figures and Tables

**Table 1:** Participant characteristics.

Work Setting	Frequency	%
Primary	23	8%
Acute	20	7%
Home	71	24%
Supportive living	39	13%
Long-term	48	16%
Community/Social	28	9%
Other	50	16%
Student	21	7%
Total	300	100%
Province	Frequency	%
Alberta	262	87%
British Columbia	5	2%
Saskatchewan	1	0%
Manitoba	3	1%
Ontario	13	4%
Prince Edward Island	2	1%
New Brunswick	2	1%
Newfoundland	3	1%
Country other than Canada	8	3%
Prefer not to answer	1	0%
Total	300	100%
Age	Frequency	%
20 or under	2	1%
21–25	11	4%
26–34	33	11%
35–44	75	25%
45–54	76	25%
55–64	88	29%
65+	14	5%
Prefer not to answer	1	0%
	300	100%
Gender	Frequency	%
Male	35	12%
Female	261	87%
Prefer not to answer	4	1%
	300	100%
Ethnicity	Frequency	%
Black	17	5%
Canadian	137	46%
Filipino	13	4%
Metis	15	5%
South Asian	11	4%
Southeast Asian	2	1%
White Caucasian	75	25%
East Asian	5	2%
Hispanic/Latinx	5	2%
Mixed Nationality	5	2%
Middle Eastern	1	0%
Prefer not to answer	14	4%
	300	100%

**Table 2:** Learners’ ratings of satisfaction with education.

Questions on Satisfaction with Education Delivery and Content	Recognizing		Communicating		Partnering		Fostering Resilience		Navigating		Changing Culture	
	$\bar{x}$ (SD)	Range (Med)	$\bar{x}$ (SD)	Range (Med)	$\bar{x}$ (SD)	Range (Med)	$\bar{x}$ (SD)	Range (Med)	$\bar{x}$ (SD)	Range (Med)	$\bar{x}$ (SD)	Range (Med)
I can apply the knowledge I learned in this module in my practice with family caregivers.	6.7 (0.7)	4–7 (7)	6.8 (0.6)	5–7 (7)	6.8 (0.5)	5–7 (7)	6.7 (0.7)	4–7 (7)	6.6 (0.6)	5–7 (7)	6.6 (0.6)	5–7 (7)
The video content helped to increase my understanding of family caregivers.	6.5 (0.9)	2–7 (7)	6.8 (0.5)	5–7 (7)	6.8 (0.6)	4–7(7)	6.7 (0.6)	4–7 (7)	6.6 (0.6)	5–7 (7)	6.7 (0.6)	5–7 (7)
This module increased my understanding of the importance of engaging and supporting family caregivers.	6.8 (0.5)	5–7 (7)	6.7 (0.6)	5–7 (7)	6.6 (0.9)	2–7 (7)	6.7 (0.6)	4–7 (7)	6.7 (0.6)	5–7 (7)	6.6 (0.8)	3–7 (7)
I found this learning to be beneficial to my practice, and I will recommend it to others.	6.6 (0.9)	2–7 (7)	6.8 (0.5)	5–7 (7)	6.8 (0.5)	5–7 (7)	6.7 (0.7)	4–7 (7)	6.6 (0.7)	5–7 (7)	6.6 (0.8)	3–7 (7)

**Table 3:** Changes **in** Learners’ Knowledge, Skills, and Attitudes.

	Paired Differences										Paired Sample Effect Sizes			
	$\mathrm{Pre}\;\bar{x}$	$\mathrm{Post}\;\bar{x}$	SMD	SD	SEM	95% CI		t	df	*p*	Standard a	Point Est	95% CI	
						Lower	Upper				Cohen’s d		Lower	Upper
Recognizing Family Caregivers	53.4	60.4	−7.0	8.5	1.2	−9.5	−4.6	−5.7	47.0	<0.001	8.5	−0.8	−1.1	−0.5
Communicating with Caregivers	59.1	61.5	−2.5	4.2	0.6	−3.8	−1.3	−4.2	47.0	<0.001	3.7	−0.7	−1.0	−0.3
Partnering with Family Caregivers	54.8	60.7	−5.9	8.0	1.1	−8.1	−3.6	−5.2	49.0	<0.001	8.0	−0.7	−1.0	−0.4
Fostering Caregivers’ Resiliency	54.6	60.1	−5.5	5.0	0.7	−7.0	−4.1	−7.7	48.0	<0.001	5.0	−1.1	−1.5	−0.7
Assisting Caregivers to Navigate	53.2	59.0	−5.8	5.9	0.9	−7.5	−4.0	−6.6	45.0	<0.001	5.9	−1.0	−1.3	−0.6
Changing Culture/Context of Care	55.1	60.0	−4.9	5.8	0.9	−6.8	−3.1	−5.3	38.0	<0.001	5.8	−0.9	−1.2	−0.5
Cohen’s d Effect Size Interpretation	Guidelines for interpreting effect size: 0.2 = small effect. 0.5 = moderate effect. 0.8 = large effect													

## Data Availability

The datasets used and/or analyzed during the current study are available from the corresponding author on reasonable request.

## Supplementary Materials

The following supporting information can be downloaded at https://www.mdpi.com/article/10.3390/healthcare13151899/s1, Supplementary File S1: Caregiver-centered care competency domains and indicators and advanced education learning outcomes. Supplementary File S2: Survey questions designed to measure pre-post changes in learners’ knowledge, skills, and attitudes. Supplementary File S3: Comparison Kirkpatrick level 2 pre-post questions, paired *T*-Tests, and Cohen’s d by competency domain. Supplementary File S4: Summary of SMART goal themes across modules.
