# Supplementary material for: Bridging the Care Gap: Integrating Family Caregiver Partnerships into Healthcare Provider Education
Source: Healthcare (Basel). 2025 Aug 4;13(15):1899. doi: 10.3390/healthcare13151899 (PMC12346123; doi:10.3390/healthcare13151899)
Supplement: Supplementary file 1 [file healthcare-13-01899-s001.zip › healthcare-3715825-supplementary.pdf]

Advanced education for healthcare providers to partner with and support family caregivers: filling a critical gap in their education

Supplementary Materials File S1: Caregiver-Centered Care Competency Domains and Indicators and Advanced Education Learning Outcomes

Learning Outcomes: Advanced Education
**Recognize the family caregiver role.** Specifically:<br>1. Describe the value of family caregivers' contributions to society and the healthcare system.<br>2. Acknowledge the positive and negative impacts of caregiving on family caregivers.<br>3. Identify and affirm the family caregiver, their roles, and their responsibilities in supporting the care recipient.<br>4. Examine the diversity of family caregiver roles, identities, and experiences.
**Partners collaboratively with family caregivers.** Specifically:<br>1. Identify the benefits of including family caregivers on the care team.<br>2. Establish collaborative relationships with family caregivers, including clarifying expectations for communication and roles.<br>3. Seek and include the family caregiver's knowledge and feedback in assessments and care planning.<br>4. Prevent, de-escalate, and repair conflict in caregiving.
**Foster the family caregiver's resilience on an ongoing basis.** Specifically:<br>1. Identify and assess the family caregiver needs, strengths, and goals on an ongoing basis.<br>2. Recognize the dynamics of the caregiver-care recipient relations and care contexts.<br>3. Enhance the family caregiver's knowledge, skills, and abilities to cope with the challenges of caring through information, education, and support.<br>4. Support the health, wellbeing, and self-care of family caregivers.
**Navigate the health and social systems with family caregivers.** Specifically:<br>1. Work collaboratively with family caregivers to access applicable supports in a timely manner.<br>2. Communicate with, and make referrals to other providers, in accordance with family caregivers' preferences.<br>3. Work with family caregivers to overcome barriers to access services, including identifying and addressing systemic issues.
**Enhance the culture and context of care.** Specifically:<br>1. Recognize that care and caregiving are affected by societal views (e.g. stigma, prejudice, discrimination).<br>2. Self-reflect upon biases to improve interactions with, and support of, family caregivers.<br>3. Model and advocate for Caregiver-Centered Care within their workplace setting.

Supplementary Materials File S2: Survey Questions designed to measure pre-post Changes in Learners' Knowledge, Skills, and Attitudes

Rated on 7-point Likert scale
Strongly Disagree, 2. Disagree, 3.Somewhat Disagree, 4. Neutral, 5. Somewhat Agree, 6. Agree, 7. Strongly Agree.

Recognizing the Caregiver Role

1. I am confident in my ability to identify family caregivers' roles and responsibilities throughout their caregiving journey.

2. I understand the importance of acknowledging family caregivers' roles, responsibilities, and the impacts of their caregiving. -

3. I understand the importance of engaging and supporting family caregivers to improve the overall caregiving experience for everyone involved (care-receiver, caregiver, care providers)

4. I know and can describe the contributions that family caregivers make to society and to the health care system. -

5. I recognize the importance of assessing caregivers' well-being in order to support their health, social connections, and quality of life.

6. I understand how caregiver well-being influences their ability to provide care for the person they support.

7. I recognize that it is important to ask family caregivers about their readiness and ability to care.

8. I am motivated to recognize and appreciate how family caregivers' roles and demands shape the support needs of family caregivers that I work with.

9. I understand all the ways that caregivers are diverse so I can be responsive to their needs.

Communicating with Family Caregivers

1. I am motivated to actively listen to family caregivers.

2. I understand how my values, beliefs, and preferences can impact my interactions with family caregivers.

3. I know how to build and maintain positive relationships with family caregivers through effective communication.

4. I understand a family caregiver's experiences and points of view are important to building a partnership with family caregivers.

5. I am motivated to be proactive in providing timely communication and follow-up with family caregivers to improve care.

6. I know how to provide clear, tailored information to family caregivers that is applicable to their needs.

7. I am determined to actively listen and speak with families to overcome challenges when coordinating care.

8. I recognize the importance of consistently documenting and sharing information with family caregivers and the healthcare teams who support them.

10. I think it is important to ask family caregivers about their willingness and ability to care.

Partnering with Family Caregivers

1. I understand the benefits of including family caregivers as part of the care team.

2. I understand what it means to collaborate with family caregivers.

3. I understand why it is important to collaborate with family caregivers early in the care trajectory.

4. I am confident in my ability to form collaborative relationships with family caregivers.

5 I know how to maintain collaborative relationships with family caregivers.

6. I understand the value of engaging with family caregivers to create and follow-up with care plans.

7. I am confident in my ability to effectively seek family caregivers' knowledge and feedback to support care planning.

8. I know what tools and strategies I can use to help prevent conflict with family caregivers.

9. I am confident in my knowledge to address conflicts that arise because of caregiving.

Fostering Family Caregivers' Resilience (Wellbeing)

1. I understand my role in supporting family caregivers' health, well-being, and ability to provide care.

2. I am committed to proactive conversations with family caregivers about their strengths and needs on an ongoing basis.

3. I am aware of the tools that can help assess and prioritize family caregivers' needs.

4. Based on an assessment of needs, I am confident in creating action plans that support the family caregiver's well-being and ability to care.

5. I can identify caregiver stress and supports to address their stressors.

6. I understand my role in facilitating supports for family caregivers.

7. I actively consider how the unique and dynamic relationship between a family caregiver and the person who needs care influences the family caregiver's support needs.

8. I understand how to explain the benefits of self-care to family caregivers.

9. I know how I can support family caregivers to engage in self-care.

10. I believe it is important to ask family caregivers about their willingness and ability to care.

Navigating the Health and Community Care Systems

1. I understand working with family caregivers to navigate health and social care systems is an important part of my care provider role.

2. I understand the types of information that can help family caregivers feel better equipped for the care journey.

3. I know how to help family caregivers navigate their journey down into manageable steps to help reduce stress.

4. I am confident in my skills to provide family caregivers with information about resources that suit their needs and preferences.

5. I understand the importance of actively expanding my knowledge of local resources and services that can support family caregivers.

6. I know how to work with family caregivers to facilitate connections with community and health care resources.

8. I know how to assist family caregivers overcome service access barriers.

8. I know how to assist family caregivers overcome service access barriers.

9. I am confident in my skills to advocate for the services and supports that family caregivers need.

Changing the Culture and Context of Care

1. I understand why self-reflection is an essential part of enhancing the care I provide for and with family caregivers.

2. I am aware of how societal biases contribute to discrimination of family caregivers.

3. I know how I can engage in self-reflection of my biases toward the care I provide for and with family caregivers.

4. I know how I can work to address my biases in my everyday practice.

5. I know what I can do to improve my interactions with family caregivers in my everyday practice.

6. I have a role in creating a culture of Caregiver-Centered Care in my workplace or discipline.

7. I am aware of ways that I can assist in promoting a culture of Caregiver-Centered Care in my workplace or discipline.

8. I feel confident in my ability to model Caregiver-Centered Care practices in my workplace or discipline.

9. I am confident that I can sustain my commitment towards improving Caregiver-Centered Care within my role.

Supplementary Materials File S3: Comparison Kirkpatrick Level 2 Pre-Post Questions, Paired T-Tests and Cohen's d by Competency Domain

Recognizing the Caregiver Role															
			**Paired Differences**									Paired Samples Effect Sizes			
	Pre Mean	Post Mean	Mean	Std. Deviation	Std. Error Mean	95% Confidence Interval of the Difference		t	df	Significance				95% Confidence Interval	
						Lower	Upper			One-Sided p	Two-Sided p	**Cohen's d**	Estimate	Lower	Upper
1. I am confident in my ability to identify family caregivers' roles and responsibilities throughout their caregiving journey.	5.69	6.53	-0.84	0.83	0.12	-1.07	-0.60	-7.10	48.00	<.001	**<.001**	0.83	-1.01	-1.36	-0.66
2. I understand the importance of acknowledging family caregivers' roles, responsibilities, and the impacts of their caregiving. -	6.16	6.76	-0.60	0.97	0.14	-0.88	-0.33	-4.38	49.00	<.001	**<.001**	0.97	-0.62	-0.92	-0.31
3. I understand the importance of engaging and supporting family caregivers to improve the overall caregiving experience for everyone involved (care-receiver, caregiver, care providers)	6.22	6.78	-0.56	1.07	0.15	-0.87	-0.26	-3.69	49.00	<.001	**<.001**	1.07	-0.52	-0.82	-0.22
4. I know and can describe the contributions that family caregivers make to society and to the health care system. -	6.78	6.78	-0.94	1.19	0.17	-1.28	-0.60	-5.61	49.00	<.001	**<.001**	1.19	-0.79	-1.11	-0.47
5. I recognize the importance of assessing caregivers' well-being in order to support their health, social connections, and quality of life.	5.94	6.71	-0.78	1.14	0.16	-1.10	-0.45	-4.76	48.00	<.001	**<.001**	1.14	-0.68	-0.99	-0.09

6. I understand how caregiver well-being influences their ability to provide care for the person they support.	6.12	6.76	-0.64	1.12	0.16	-0.96	-0.32	-4.04	49.00	<.001	**<.001**	1.12	-0.57	-0.87	-0.36
7. I recognize that it is important to ask family caregivers about their readiness and ability to care.	5.88	6.76	-0.88	1.27	0.18	-1.24	-0.51	-4.84	48.00	<.001	**<.001**	1.27	-0.69	-1.00	-0.27
8. I am motivated to recognize and appreciate how family caregivers' roles and demands shape the support needs of family caregivers that I work with.	6.12	6.78	-0.66	1.15	0.16	-0.99	-0.33	-4.05	49.00	<.001	**<.001**	1.15	-0.57	-0.87	-0.37
9. I understand all the ways that caregivers are diverse so I can be responsive to their needs.	5.68	6.74	-1.06	1.20	0.17	-1.40	-0.72	-6.24	49.00	<.001	**<.001**	1.20	-0.88	-1.21	-0.27

**Communicating with Family Caregivers**															
	Paired Differences								df	Significance		Paired Samples Effect Sizes			
	Pre Mean	Post Mean	Mean	Std. Deviation	Std. Error Mean	95% Confidence Interval of the Difference		t		One-Sided p	Two-Sided p	Standardized	Point Estimate	95% Confidence Interval	
						Lower	Upper					**Cohen's d**		Lower	Upper
1. I am motivated to actively listen to family caregivers.	6.72	6.86	-0.14	0.45	0.06	-0.27	-0.01	-2.19	49.00	0.02	**0.03**	0.45	**-0.31**	-0.59	-0.02
2. I understand how my values, beliefs, and preferences can impact my interactions with family caregivers.	6.64	6.84	-0.20	0.61	0.09	-0.37	-0.03	-2.33	49.00	0.01	**0.02**	0.61	**-0.33**	-0.61	-0.04

3. I know how to build and maintain positive relationships with family caregivers through effective communication.	6.24	6.78	-0.54	0.84	0.12	-0.78	-0.30	-4.56	49.00	<.001	**<.001**	0.84	**-0.64**	-0.95	-0.34
4. I understand a family caregiver's experiences and points of view are important to building a partnership with family caregivers.	6.59	6.90	-0.31	0.59	0.08	-0.47	-0.14	-3.67	48.00	<.001	**<.001**	0.59	**-0.52**	-0.82	-0.22
5. I am motivated to be proactive in providing timely communication and follow-up with family caregivers to improve care.	6.64	6.90	-0.26	0.53	0.08	-0.41	-0.11	-3.49	49.00	<.001	**0.00**	0.53	**-0.49**	-0.79	-0.20
6. I know how to provide clear, tailored information to family caregivers that is applicable to their needs.	6.24	6.70	-0.46	0.76	0.11	-0.68	-0.24	-4.27	49.00	<.001	**<.001**	0.76	**-0.60**	-0.90	-0.30
7. I am determined to actively listen and speak with families to overcome challenges when coordinating care.	6.54	6.82	-0.28	0.64	0.09	-0.46	-0.10	-3.09	49.00	0.00	**0.00**	0.64	**-0.44**	-0.73	-0.15
8. I recognize the importance of consistently documenting and sharing information with family caregivers and the healthcare teams who support them.	6.62	6.86	-0.24	0.59	0.08	-0.41	-0.07	-2.87	49.00	0.00	**0.01**	0.59	**-0.41**	-0.69	-0.12
10. I think it is important to ask family caregivers about their willingness and ability to care.	6.64	6.86	-0.22	0.58	0.08	-0.39	-0.06	-2.67	49.00	0.01	**0.01**	0.58	**-0.38**	-0.66	-0.09
Mean **Partnering with Family Caregivers**															
			Paired Differences					t	df	Significance		Paired Samples Effect Sizes			
			Mean	Std. Deviation	Std. Error Mean	95% Confidence Interval of the Difference				One-Sided p	Two-Sided p	Standardizera	Point Estimate	95% Confidence Interval	

	Pre-Mean	Post-Mean				Lower	Upper					Cohen's d		Lower	Upper
1. I understand the benefits of including family caregivers as part of the care team.	6.50	6.84	-0.34	1.00	0.14	-0.63	-0.06	-2.40	49.00	0.01	**0.02**	1.00	**-0.34**	-0.62	-0.05
2. I understand what it means to collaborate with family caregivers.	6.38	6.84	-0.46	0.99	0.14	-0.74	-0.18	-3.27	49.00	<.001	**0.00**	0.99	**-0.46**	-0.75	-0.17
3. I understand why it is important to collaborate with family caregivers early in the care trajectory.	6.40	6.82	-0.42	1.01	0.14	-0.71	-0.13	-2.94	49.00	0.00	**0.01**	1.01	**-0.42**	-0.70	-0.12
4. I am confident in my ability to form collaborative relationships with family caregivers.	6.04	6.65	-0.61	1.04	0.15	-0.91	-0.31	-4.13	48.00	<.001	**<.001**	1.04	**-0.59**	-0.89	-0.28
5 I know how to maintain collaborative relationships with family caregivers.	5.98	6.72	-0.74	1.12	0.16	-1.06	-0.42	-4.67	49.00	<.001	**<.001**	1.12	**-0.66**	-0.96	-0.35
6. I understand the value of engaging with family caregivers to create and follow-up with care plans.	6.35	6.80	-0.45	0.98	0.14	-0.73	-0.17	-3.21	48.00	0.00	**0.00**	0.98	**-0.46**	-0.75	-0.16
7. I am confident in my ability to effectively seek family caregivers’ knowledge and feedback to support care planning.	6.06	6.72	-0.66	0.96	0.14	-0.93	-0.39	-4.86	49.00	<.001	**<.001**	0.96	**-0.69**	-0.99	-0.38
8. I know what tools and strategies I can use to help prevent conflict with family caregivers.	5.54	6.64	-1.10	1.23	0.17	-1.45	-0.75	-6.31	49.00	<.001	**<.001**	1.23	**-0.89**	-1.22	-0.56
9. I am confident in my knowledge to address conflicts that arise because of caregiving.	5.54	6.64	-1.10	1.17	0.17	-1.43	-0.77	-6.68	49.00	<.001	**<.001**	1.17	**-0.94**	-1.28	-0.61
**Fostering Family Caregivers' Resilience (Wellbeing)**															

	Paired Differences							t	df	Significance		Paired Samples Effect Sizes			
	Pre Mean	Post Mean	Mean	Std. Deviation	Std. Error Mean	95% Confidence Interval of the Difference				One-Sided p	Two-Sided p	Standardizer a	Point Estimate	95% Confidence Interval	
						Lower	Upper					**Cohen's d**		Lower	Upper
1 I understand my role in supporting family caregivers' health, well-being, and ability to provide care.	6.30	6.68	-0.38	0.70	0.10	-0.58	-0.18	-3.86	49.00	<.001	**<.001**	0.70	**-0.55**	-0.84	-0.25
2 I am committed to proactive conversations with family caregivers about their strengths and needs on an ongoing basis.	6.46	6.78	-0.32	0.59	0.08	-0.49	-0.15	-3.86	49.00	<.001	**<.001**	0.59	**-0.55**	-0.84	-0.25
3. I am aware of the tools that can help assess and prioritize family caregivers' needs.	5.56	6.70	-1.14	1.29	0.18	-1.51	-0.77	-6.23	49.00	<.001	**<.001**	1.29	**-0.88**	-1.21	-0.55
4. Based on an assessment of needs, I am confident in creating action plans that support the family caregiver's well-being and ability to care.	5.84	6.60	-0.76	0.82	0.12	-0.99	-0.53	-6.54	49.00	<.001	**<.001**	0.82	**-0.92**	-1.25	-0.59
5. I can identify caregiver stress and supports to address their stressors.	6.02	6.74	-0.72	0.83	0.12	-0.96	-0.48	-6.11	49.00	<.001	**<.001**	0.83	**-0.86**	-1.19	-0.54
6. I understand my role in facilitating supports for family caregivers.	6.14	6.72	-0.58	0.76	0.11	-0.80	-0.36	-5.41	49.00	<.001	**<.001**	0.76	**-0.77**	-1.08	-0.45
7. I actively consider how the unique and dynamic relationship between a family caregiver and the person who needs care influences the family caregiver's support needs.	6.34	6.70	-0.36	0.63	0.09	-0.54	-0.18	-4.03	49.00	<.001	**<.001**	0.63	**-0.57**	-0.87	-0.27
8. I understand how to explain the benefits of self-care to family caregivers.	6.16	6.61	-0.45	0.68	0.10	-0.64	-0.25	-4.63	48.00	<.001	**<.001**	0.68	**-0.66**	-0.97	-0.35

9. I know how I can support family caregivers to engage in self-care.	5.88	6.60	-0.72	0.73	0.10	-0.93	-0.51	-6.98	49.00	<.001	**<.001**	0.73	**-0.99**	-1.32	-0.65
10. I believe it is important to ask family caregivers about their willingness and ability to care.	6.52	6.80	-0.28	0.61	0.09	-0.45	-0.11	-3.26	49.00	0.00	**0.00**	0.61	**-0.46**	-0.75	-0.17
**Navigating the Health and Community Care Systems**															
			Paired Differences					t	df	Significance		Paired Samples Effect Sizes			
			Mean	Std. Deviation	Std. Error Mean	95% Confidence Interval of the Difference				One-Sided p	Two-Sided p	Standardizer a	Point Estimate	95% Confidence Interval	
	Pre Mean	Pre Mean				Lower	Upper					**Cohen's d**		Lower	Upper
1. I understand working with family caregivers to navigate health and social care systems is an important part of my care provider role.	6.52	6.72	-0.20	0.70	0.10	-0.40	0.00	-2.02	49.00	0.02	**0.05**	0.70	**-0.29**	-0.57	0.00
2. I understand the types of information that can help family caregivers feel better equipped for the care journey.	5.96	6.67	-0.71	0.96	0.14	-0.99	-0.44	-5.22	48.00	<.001	**<.001**	0.96	**-0.75**	-1.06	-0.43
3. I know how to help family caregivers navigate their journey down into manageable steps to help reduce stress.	5.78	6.55	-0.78	0.90	0.13	-1.03	-0.52	-6.06	48.00	<.001	**<.001**	0.90	**-0.87**	-1.19	-0.53
4. I am confident in my skills to provide family caregivers with information about resources that suit their needs and preferences.	5.74	6.52	-0.78	0.84	0.12	-1.02	-0.54	-6.57	49.00	<.001	**<.001**	0.84	**-0.93**	-1.26	-0.59
5. I understand the importance of actively expanding my knowledge of local resources and services that can support family caregivers.	6.48	6.78	-0.30	0.68	0.10	-0.49	-0.11	-3.13	49.00	0.00	**0.00**	0.68	**-0.44**	-0.73	-0.15

6. I know how to work with family caregivers to facilitate connections with community and health care resources.	5.92	6.54	-0.62	0.99	0.14	-0.90	-0.34	-4.44	49.00	<.001	**<.001**	0.99	**-0.63**	-0.93	-0.32
8. I know how to assist family caregivers overcome service access barriers.	5.57	6.49	-0.92	0.98	0.14	-1.20	-0.64	-6.59	48.00	<.001	**<.001**	0.98	**-0.94**	-1.28	-0.60
8. I know how to assist family caregivers overcome service access barriers.	5.57	6.41	-0.84	0.97	0.14	-1.11	-0.56	-6.07	48.00	<.001	**<.001**	0.97	**-0.87**	-1.19	-0.54
9. I am confident in my skills to advocate for the services and supports that family caregivers need.	5.74	6.56	-0.82	0.85	0.12	-1.06	-0.58	-6.82	49.00	<.001	**<.001**	0.85	**-0.97**	-1.30	-0.63
**Changing the Culture and Context of Care**															
			Paired Differences					t	df	Significance					
			Mean	Std. Deviation	Std. Error Mean	95% Confidence Interval of the Difference				One-Sided p	Two-Sided p	Standardized	Point Estimate	95% Confidence Interval	
	Mean					Lower	Upper					Cohen's d		Lower	Upper
1. I understand why self-reflection is an essential part of enhancing the care I provide for and with family caregivers.	6.53	6.71	-0.18	0.70	0.10	-0.38	0.02	-1.84	48.00	0.04	0.07	0.69	**-0.26**	-0.55	0.02
2. I am aware of how societal biases contribute to discrimination of family caregivers.	6.24	6.72	-0.48	0.89	0.13	-0.73	-0.23	-3.83	49.00	<.001	**<.001**	0.89	**-0.54**	-0.84	-0.24
3. I know how I can engage in self-reflection of my biases toward the care I provide for and with family caregivers.	5.98	6.57	-0.59	0.93	0.13	-0.86	-0.32	-4.44	48.00	<.001	**<.001**	0.93	**-0.63**	-0.94	-0.32
4. I know how I can work to address my biases in my everyday practice.	5.96	6.53	-0.57	0.79	0.11	-0.80	-0.34	-5.06	48.00	<.001	**<.001**	0.79	**-0.72**	-1.04	-0.41

5. I know what I can do to improve my interactions with family caregivers in my everyday practice.	6.00	6.66	-0.66	0.82	0.12	-0.89	-0.43	-5.67	49.00	<.001	**<.001**	0.82	**-0.80**	-1.12	-0.48
6. I have a role in creating a culture of Caregiver-Centered Care in my workplace or discipline.	6.38	6.72	-0.34	0.94	0.13	-0.61	-0.07	-2.56	49.00	0.01	**0.01**	0.94	**-0.36**	-0.65	-0.07
7. I am aware of ways that I can assist in promoting a culture of Caregiver-Centered Care in my workplace or discipline.	6.10	6.69	-0.58	1.03	0.15	-0.88	-0.29	-3.93	47.00	<.001	**<.001**	1.03	**-0.57**	-0.87	-0.26
8. I feel confident in my ability to model Caregiver-Centered Care practices in my workplace or discipline.	6.00	6.64	-0.64	0.78	0.11	-0.86	-0.42	-5.83	49.00	<.001	**<.001**	0.78	**-0.83**	-1.14	-0.50
9. I am confident that I can sustain my commitment towards improving Caregiver-Centered Care within my role.	6.25	6.66	-0.41	0.73	0.11	-0.63	-0.19	-3.74	43.00	<.001	**<.001**	0.73	**-0.56**	-0.88	-0.24
Cohen's d Effect Size Interpretation	The general guidelines for interpreting the effect size are as follows: 0.2 = small effect. 0.5 = moderate effect. 0.8 = large effect														

Supplementary File S4: Summary of SMART Goal Themes Across Modules

Module	Theme	Description	Representative Examples
Recognizing the Family Caregiver Role	Affirmation and Acknowledgement	Recognizing, affirming, and documenting caregiver contributions and sacrifices in care settings	“Identify and document family caregivers.” “Acknowledge each caregiver’s sacrifices at care conferences.”
Communicating with Family Caregivers	Empathic Communication	Using communication frameworks (e.g., OARS, H.E.A.R.T.) to strengthen caregiver relationships	“Practice OARS in daily work.” “Use H.E.A.R.T. to deescalate tension with caregivers.”
Partnering with Family Caregivers	Collaborative Care Planning	Building trust and engaging caregivers as equal partners in decision-making and care delivery	“Share care plans with caregivers.” “Ensure home care and caregivers communicate as a team.”
Fostering Caregiver Resilience	Strengths-Based Support	Promoting caregiver wellbeing through listening, validation, goal setting, and follow-up	“Use CSNAT-I to co-develop caregiver goals.” “Acknowledge caregivers’ strengths during transitions.”
Assisting Caregivers to Navigate Systems	Resource Connection and Guidance	Supporting caregivers’ system navigation and access to information and services	“Develop a contact bank for caregiver referrals.” “Follow up after resource referrals to assess progress.”
Changing Culture and Context of Care	Leadership and Culture Change	Challenging bias, advocating for caregiver-centered practices, and modeling inclusive behaviors	“Reflect on unconscious bias daily.” “Lead change by sharing caregiver-centered care with organizational leaders.”
